# Implementing a centralised pharmacovigilance service in a non-commercial setting in the United Kingdom

**DOI:** 10.1186/1745-6215-14-171

**Published:** 2013-06-12

**Authors:** Eleanor M Dinnett, Sharon Kean, Elizabeth P Tolmie, Elizabeth S Ronald, Allan Gaw

**Affiliations:** 1 Glasgow Clinical Trials Unit, Robertson Centre for Biostatistics, Boyd Orr Building, Glasgow G12 8QQ, UK; 2 College of Medical, Veterinary and Life Sciences, University of Glasgow, Glasgow, UK; 3 Scottish Stroke Research Network, Walton Building, Glasgow Royal Infirmary, Glasgow, UK; 4 Associate Director for Education Quality Standards, NIHR-CRN, University of Leeds, Leeds, UK

**Keywords:** Pharmacovigilance, Safety reporting, Public sector, Non-commercial, Service set-up

## Abstract

The implementation of a pharmacovigilance service compliant with the legal and regulatory responsibilities of clinical trial sponsors presents particular challenges for sponsors in a non-commercial setting.

In this paper we examine these challenges in detail. We identify and discuss the key steps in the development of a pharmacovigilance service within a public health service and university setting in the United Kingdom. We describe how we have established a central Pharmacovigilance Office with dedicated staff and resources within our organisation. This office is supported by an electronic pharmacovigilance reporting infrastructure developed to facilitate the receipt and processing of safety information, the onward reporting in compliance with legislation and the provision of sponsor institution oversight of clinical trial participant safety. An education and training programme has also been set up to ensure that all relevant staff in the organisation are fully aware of the pharmacovigilance service and are appropriately trained in its use.

We discuss possible alternatives to this approach and why we consider our solution to be the most appropriate to ensure that a non-commercial sponsor organisation and investigators are operating in a fully compliant way.

## Review

### Background

Pharmacovigilance is defined by the World Health Organization as the pharmacological science relating to the detection, assessment, understanding and prevention of adverse effects, particularly long-term and short-term side effects, of medicines [1]. In order to comply with European and UK regulatory requirements these activities must be carried out for both authorised medicinal products and for those used within a clinical research programme. Any organisation taking on the role of sponsor of clinical trials of investigational medicinal products (CTIMPs) must put in place arrangements for safety reporting as part of a pharmacovigilance programme.

Within the pharmaceutical industry, pharmacovigilance is a major component of clinical trial conduct and attracts considerable resources. Within the public sector, pharmacovigilance has in recent years become an equally important concern especially across the European Union (EU) member states following the implementation of the EU Clinical Trials Directive (2001/20/EC) [2] and its transposition into UK law by The Medicines for Human Use (Clinical Trials) Regulations 2004 [3] thereby setting out specific requirements for pharmacovigilance in CTIMPs. These regulations describe pharmacovigilance as ‘the recording and reporting of adverse events and reactions to medicinal products being used in a clinical trial’ with Regulations 32 to 35 detailing the requirements.

Within the public sector the provision of a pharmacovigilance service to support clinical trials and to fulfil the legal and regulatory responsibilities of sponsors carries a number of particular challenges largely because of the limitation of resources and available expertise. In this paper we examine these challenges in detail, describe how we have established a robust pharmacovigilance service within a public health service/university setting and discuss the lessons we have learned.

### Requirements of a pharmacovigilance service

In order to establish a pharmacovigilance and safety reporting service it is first necessary to understand the requirements of such a service. The responsibilities for pharmacovigilance are laid out within a range of key documents including the EU Clinical Trials Directive [2], The Medicines for Human Use (Clinical Trials) Regulations 2004 [3], ICH-GCP E6 [4] and governance guidance documents such as the UK Research Governance Frameworks [5,6].

Most recently these responsibilities have been defined in a new composite guidance document from the European Commission [7] as detailed in Table 1.

**Table 1:** **Summary of safety reporting responsibilities**[7]

***Investigator responsibilities***	***Sponsor responsibilities***
Report serious adverse events to the sponsor as per the protocol	Keep detailed records of all adverse events which are reported by the investigator or investigators
Report certain non-serious adverse events and/or laboratory abnormalities to the sponsor as per the protocol	Report suspected unexpected serious adverse reactions (SUSARs) to the national competent authority and the Ethics Committee
	Inform investigators of SUSARs
	Submit an annual safety report to the national competent authority and the Ethics Committee
	Continuously weigh anticipated benefits and risks of the clinical trial which includes ongoing safety evaluation of IMPs

The responsibilities of the sponsor with respect to pharmacovigilance and safety reporting are clearly extensive and can only be fulfilled by creating a robust reporting system backed up with education, training and oversight of the process. The challenge is how to achieve such a system, which will be acceptable to any auditor or inspector from both the public and private sectors.

**Figure 1:**
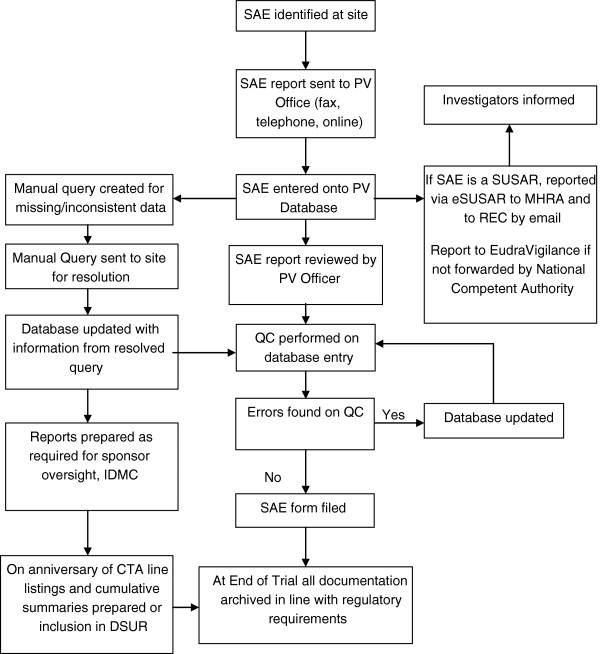
**Flowchart of SAE data.** CTA, clinical trial authorisation; DSUR, Development safety update report; IDMC, Independent data monitoring committee; MHRA, Medicines and healthcare products regulatory agency; PV, pharmacovigilance; QC, quality control; REC, Research ethics committee; SAE, Serious adverse event; SAR, Serious adverse reaction; SUSAR, suspected unexpected serious adverse reaction.

The development of an effective pharmacovigilance service in a non-commercial setting is a complex process involving a number of different stakeholders and their competing agendas and is often built on existing, sometimes piecemeal, services. The development of our service was to some extent the product of local factors, but much of what we learned in the process is applicable to others. We have identified the following seven key steps in the development of our service and will discuss each in turn:

1. Get stakeholders together

2. Review existing services

3. Define the process

4. Translate the process into standard operating procedures

5. Decide how to operationalise these and what resources are needed

6. Implement training and education of research staff

7. Maintain oversight

1. Get stakeholders together: An effective pharmacovigilance service requires input from a number of key partners in the process. These may include the local NHS Research and Development Office, Hospital Pharmacy, local Clinical Research Facilities, Data Management services and Universities. To facilitate this interaction a pharmacovigilance group should be established with representation from each stakeholder. This group will serve as the operational committee implementing and overseeing any new service.

2. Review existing services: The first task of the pharmacovigilance group is to review any existing pharmacovigilance and safety reporting services. In most circumstances clinical research will already be ongoing in an institution and there may be departments or even individuals who have already developed effective systems. Often, however, there will be no coherent service or any services that do exist may be fragmentary, specific to individual studies and perhaps even conflicting. It is important, however, to recognise examples of good practice and if possible incorporate them into any new service.

3. Define the process: Assuming that a new pharmacovigilance service is required, the next step is to define the nature of this. While pharmacovigilance is defined as a requirement in UK law there is limited guidance on how to fulfil this responsibility. It is important that the pharmacovigilance group has expertise in pharmacovigilance and safety reporting in practice and should develop, in the first instance, a flow chart of adverse event reporting and processing that may be endorsed by the group as a whole. An example of such a flowchart is shown in Figure 1. The exact nature of this process will depend on the scale of the operation envisaged and available resources.

4. Translate the process into standard operating procedures (SOPs): Once the process has been agreed, it must be broken down into its component parts and defined in the form of SOPs. These SOPs should be signed off and managed in the same way as all other institutional SOPs.

5. Decide how to operationalise these and what resources are needed: The pharmacovigilance process detailed in the SOPs will inevitably require resources for its implementation. This may involve the identification and support of specific staff who will serve as the pharmacovigilance team. It may also require the identification of space to serve as a Pharmacovigilance Office. Alternatively, some institutions will effectively delegate much of the delivery of pharmacovigilance activities to individual investigators. If so, it must be remembered that the responsibility remains with the sponsoring institution. Again the scale of the required resources will be dependent on the number of clinical trials undertaken by an institution and their anticipated pharmacovigilance needs. Based on this evaluation it may be apparent that the amount of pharmacovigilance activity anticipated does not justify a dedicated staff or office. If so, the group may decide to outsource the pharmacovigilance service rather than create its own. A number of organisations are currently able to provide different aspects of such a service. These vendors include commercial clinical research organisations, but also may include public sector groups such as universities and health service groups, who have developed robust systems for their own needs and are now in a position to take on a pharmacovigilance and/or safety reporting service provision for others.

6. Implementing training and education of research staff: The institution must ensure that all relevant staff are fully aware of the service and are appropriately trained in its use. Most institutions will provide researchers with Good Clinical Practice (GCP) training. Often this will include a generic pharmacovigilance component, but may lack the specifics required for an investigator to identify, assess, report and action any Serious Adverse Event (SAE) report using the local systems and in line with regulatory requirements. If this is the case additional education and training will be needed for those staff involved in CTIMPs and may be provided by the Pharmacovigilance Office.

7. Maintain oversight: Irrespective of how the service is provided it is essential that the sponsor institution retains an oversight function to ensure that their legal responsibilities for pharmacovigilance are met. Part of this oversight will be ensuring that any duties delegated to investigators or third parties are delivered in a timely fashion, and that the overall process is kept under review to identify any problems. Pharmacovigilance SOPs, like all SOPs, should be reviewed and if necessary revised, on a regular basis. The pharmacovigilance group will have a key role in this as well as defining any developments required in the education and training of staff.

### Implementation of our pharmacovigilance service

The provision of a pharmacovigilance service presents a number of challenges in the public sector. Our responses to these challenges have evolved and developed over the last 8 years and involve several interlinked components. Key to our strategy has been establishing a central Pharmacovigilance Office with dedicated staff and resources to service a wide range of studies sponsored by our institutions. Organisationally this office sits at the junction between the NHS and the University and draws expertise from both. The provision of pharmacovigilance support requires a mixture of medical, administrative and IT skills all of which we have concentrated in our Pharmacovigilance Office. While major elements of safety reporting in CTIMPs will be delivered by members of investigative teams in the field we believe that without the support of a centralised office the pharmacovigilance responsibilities of the sponsor would not be adequately fulfilled. The Pharmacovigilance Office has supported a number of trials since it was established in 2009. We are currently supporting 10 active CTIMPs for our sponsor organisation with a further 22 CTIMPs now completed. This includes a variety of types of trials from single site to multicentre international trials .In addition the electronic reporting system has been or is currently used in a further 13 trials for a number of commercial and non-commercial sponsors. Our office has a medically qualified Pharmacovigilance Officer. While not every organisation may have access to such an individual we believe that having a medically qualified officer with clinical trial experience has greatly enhanced our service. Other members of the team including the administrative and IT support staff should also have a background in clinical research as this will facilitate a more efficient and user friendly service. It is important that the Pharmacovigilance Office is not simply viewed as another bureaucratic hurdle for investigators to leap, but rather as an approachable and useful service. This service includes support during the trial, but also extends to advice before the trial with protocol design and any study specific reporting requirements, for example, in studies using Advanced Therapy Investigational Medicinal Products (ATIMPs) which, although governed by the same legislation as other clinical trials, have additional reporting requirements [8]. Support during a trial includes the review and triaging of all SAE reports received against the information already known of the expected adverse effects of the IMP in order to identify any suspected unexpected serious adverse reactions (SUSARs) which will require expedited reporting to regulatory authorities. The Pharmacovigilance Office also supports the preparation and submission of annual safety reports as required by legislation. Advice is also provided after the close of the trial regarding the requirements for the archiving of pharmacovigilance documents in line with regulatory and any study specific requirements. When planning our office set-up, its location, structure and staffing were all taken in to account to ensure the best use of existing resources and the future needs of the service.

To streamline the service, an electronic pharmacovigilance reporting infrastructure was developed. This incorporates a system for the remote capture of SAE information via a secure web portal. Alternatively investigators can fax reports into a central fax server. Incorporated into this system are automatic email alerts to pharmacovigilance staff when an SAE report is received facilitating timely review of the event. The data are held in a central database and the system can produce individual case safety reports in various layouts, including the Council for International Organizations of Medical Sciences format. The database conforms to the requirements of the relevant European guidance on the electronic submission of safety reports [9] and can therefore facilitate direct reporting to the EudraVigilance Clinical Trial Module. The system also facilitates the generation of metrics and line listings that can be used for the production of reports required for sponsor or management oversight, review by Independent Data Monitoring Committee (IDMC) or for submission of annual safety reports in the format of a Development Safety Update Report (DSUR) [10] to regulatory authorities, research ethics committees or collaborators.

In line with internal quality management, sponsor organisation processes and ISO 9001:2008 accreditation we are continually reviewing and improving the electronic pharmacovigilance system and its supporting documentation. Feedback from external audits and inspections has also led to improvements to the system. The system has been GCP inspected by the UK Competent Authority on five occasions and is audited by other sponsor organisations on a regular basis with no major findings. Our service is underpinned by an education and training programme that is available to all researchers and their support staff. Without appropriate training any pharmacovigilance service will fail. Field staff have to be aware of the pharmacovigilance office and of the services it provides and must be familiar with the institution’s SOPs regarding safety reporting. To achieve this we have supplemented our standard GCP course with standalone pharmacovigilance workshops and study specific training designed specifically for those undertaking CTIMPs.

## Conclusions

While our approach has been successful it is not the only potential solution to the problem. In Glasgow, as elsewhere, we could have decided to outsource the pharmacovigilance service to commercial organisations. This would have been costly and whatever the level of service provided, the legislation is clear that the delegation of tasks does not remove the ultimate responsibility of the sponsor for the conduct of the clinical trial in accordance with the applicable legislation. As such, an extensive oversight mechanism would have had to be established, which in itself would have had resource implications. Cost consideration is always a high priority in the public sector and our decision not to outsource was primarily a financial one.

Because we are working in a constantly changing regulatory landscape we need to be able to respond to new legislation and governance systems. Within the UK we can expect major changes in the governance of clinical research following the publication of Academy of Medical Sciences Report [11] and the Proposal for a Regulation of the European Parliament and of the Council on clinical trials on medicinal products for human use, and repealing Directive 2001/20/EC [12]. Having a dedicated pharmacovigilance staff who can keep abreast of these changes and amend their systems accordingly will ensure that the sponsor organisation and investigators are operating in a fully compliant way.

## Abbreviations

ATIMP: Advanced Therapy Investigational Medicinal Product; CTIMP: Clinical trial of an investigational medicinal product; DSUR: Development Safety Update Report; IDMC: Independent Data Monitoring Committee; NHS: National Health Service; SOP: Standard operating procedure; GCP: Good Clinical Practice; SAE: Serious Adverse Event; SAR: Serious Adverse Reaction; SUSAR: Suspected unexpected serious adverse reaction; ICH: International Conference on Harmonisation.

## Competing interests

The authors declare that they have no competing interests.

## Authors’ contributions

All authors were involved in the establishment of the Pharmacovigilance Service. EMD, AG, and SK drafted the manuscript. ET and ESR reviewed the manuscript. All authors read and approved the final manuscript.
